# Supplementary material for: Integrative approaches to a revision of the liverwort in genus Aneura (Aneuraceae, Marchantiophyta) from Thailand
Source: PeerJ. 2023 Oct 24;11:e16284. doi: 10.7717/peerj.16284 (PMC10607200; doi:10.7717/peerj.16284)
Supplement: Figure S1 — Outgroups, including Riccardia and Lobatiriccardia, were removed before the species delimitation. [file peerj-11-16284-s001.docx]

**Figure S1 Lineages through time (LTT) plot from an ultrametric tree.** Outgroups, including *Riccardia* and *Lobatiriccardia*, were removed before the species delimitation.


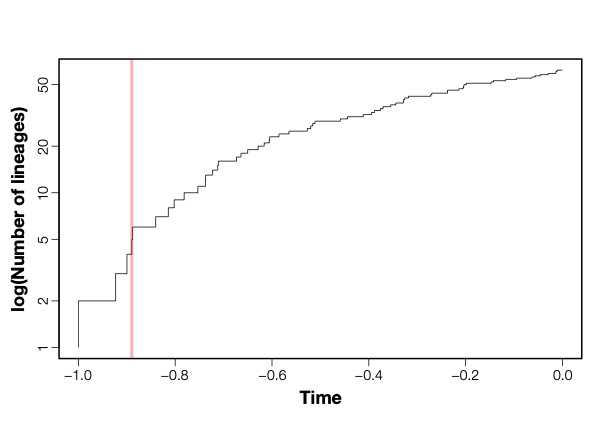


*Red line showed the threshold from the “single” method of the GMYC analysis*
