# Supplementary material for: Integrative approaches to a revision of the liverwort in genus Aneura (Aneuraceae, Marchantiophyta) from Thailand
Source: PeerJ. 2023 Oct 24;11:e16284. doi: 10.7717/peerj.16284 (PMC10607200; doi:10.7717/peerj.16284)
Supplement: Figure S2 — Quantitative characters were recognized by non-parametric Wilcoxon Rank Sum and Signed Rank Tests, while the qualitative characters were recognized by Pearson’s Chi-squared test. [file peerj-11-16284-s002.docx]

**Figure S2 Morphological difference among genetic clades of GMYC with single threshold within *Aneura* species from Thailand.** Quantitative characters were recognized by non-parametric Wilcoxon Rank Sum and Signed Rank Tests, while the qualitative characters were recognized by Pearson's Chi-squared test.

*
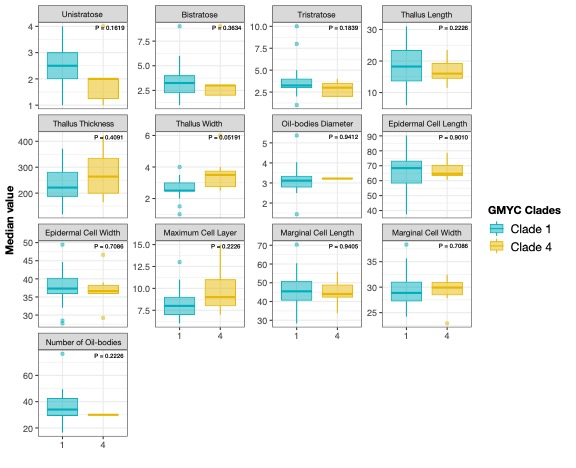
*

*R boxplot continuous characters with p-value*

*
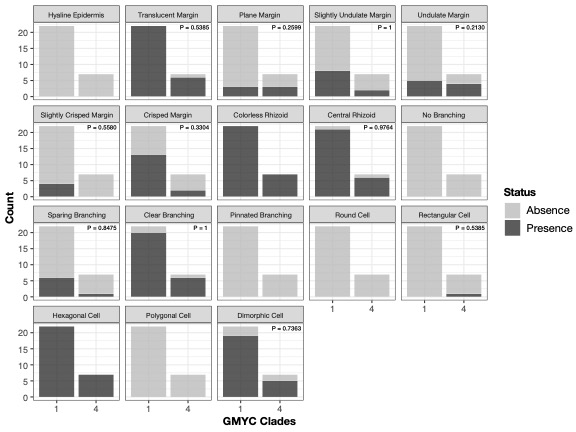
*

*R barplot discrete characters with p-value*
