# Supplementary material for: Integrative approaches to a revision of the liverwort in genus Aneura (Aneuraceae, Marchantiophyta) from Thailand
Source: PeerJ. 2023 Oct 24;11:e16284. doi: 10.7717/peerj.16284 (PMC10607200; doi:10.7717/peerj.16284)
Supplement: Table S1 [file peerj-11-16284-s003.docx]

**Table S1 Morphological and anatomical characters in this study**

| **Characters** | **Formats** | **Description** | **Views** |
| --- | --- | --- | --- |
| **Quantitative traits** | | | |
| Unistratose | Integer | Counting the number of 1-layer cells | Cross section |
| Bistratose | Integer | Counting the number of 2-layer cells | Cross section |
| Tristratose | Integer | Counting the number of 3-layer cells | Cross section |
| Maximum cell layer | Integer | Counting the highest cell layer from each thallus | Cross section |
| Number of oil-bodies | Integer | Counting the number of oil-bodies in cells | Dorsal side |
| Oil-bodies diameter | Decimal | Measuring the diameter of oil-bodies in cells | Dorsal side |
| Thallus width | Decimal | Measuring width from mature gametophyte | Dorsal side |
| Thallus length | Decimal | Measuring length from mature gametophyte | Dorsal side |
| Thallus thickness | Decimal | Measuring thickness from section of mature gametophyte | Dorsal side |
| Epidermal cell width | Decimal | Measuring cell width from median area of thallus | Dorsal side |
| Epidermal cell length | Decimal | Measuring cell length from median area of thallus | Dorsal side |
| Marginal cell width | Decimal | Measuring cell width from marginal area of thallus | Dorsal side |
| Marginal cell length | Decimal | Measuring cell length from marginal area of thallus | Dorsal side |
| **Qualitative traits** | | | |
| Hyaline epidermis | Binomial | (0) Epidermal layer occurs as chlorophyllous cells  (1) Epidermal layer occurs as hyaline cells | Cross section |
| Small oil-bodies | Binomial | (0) Oil-bodies are larger than plastid  (1) Oil-bodies are distinctly smaller than plastid | Dorsal side |
| Colorless oil-bodies | Binomial | (0) Oil-bodies are colored  (1) Oil-bodies are colorless | Dorsal side |
| Translucent margin | Binomial | (0) Marginal thallus are opaque or translucent less than 5 cells  (1) Marginal thallus are translucent about 5 cells | Dorsal side |
| Plane thallus | Binomial | (0) Absence  (1) Presence | Dorsal side |
| Slightly undulate thallus | Binomial | (0) Absence with small loop  (1) Presence | Dorsal side |
| Undulate thallus | Binomial | (0) Absence with clear loop  (1) Presence | Dorsal side |
| Slightly crisped thallus | Binomial | (0) Absence with small loop  (1) Presence | Dorsal side |
| Crisped thallus | Binomial | (0) Absence with clear loop  (1) Presence | Dorsal side |
| Colorless rhizoid | Binomial | (0) Rhizoids are colored  (1) Rhizoids are colorless | Ventral side |
| Central rhizoid | Binomial | (0) Rhizoids spread regularly on main thallus  (1) Rhizoids attach on the middle of main thallus | Ventral side |
| Unbranched thallus | Binomial | (0) Main thallus has a branching  (1) Main thallus has no branch | Dorsal side |
| Sparing branched thallus | Binomial | (0) Lateral branches are absent or larger than main thallus  (1) Lateral branches are smaller than main thallus | Dorsal side |
| Clear branched thallus | Binomial | (0) Lateral branches are absent or smaller than main thallus  (1) Lateral branches are equal to main thallus | Dorsal side |
| Pinnated branched thallus | Binomial | (0) Absence  (1) Presence | Dorsal side |
| Round cell | Binomial | (0) Absence  (1) Presence | Dorsal side |
| Rectangular cell | Binomial | (0) Absence  (1) Presence | Dorsal side |
| Hexagonal cell | Binomial | (0) Absence  (1) Presence | Dorsal side |
| Polygonal cell | Binomial | (0) Absence  (1) Presence | Dorsal side |
| Dimorphic cell | Binomial | (0) Marginal cells shape similarly to median cells  (1) Marginal cells shape differently from median cells | Dorsal side |
