# Supplementary material for: Integrative approaches to a revision of the liverwort in genus Aneura (Aneuraceae, Marchantiophyta) from Thailand
Source: PeerJ. 2023 Oct 24;11:e16284. doi: 10.7717/peerj.16284 (PMC10607200; doi:10.7717/peerj.16284)
Supplement: Table S2 — All bold accession numbers represent sequences generated from this study. [file peerj-11-16284-s004.docx]

**Table S2 Voucher numbers with geographic details and GenBank accession numbers for the sequences in this study.** All bold accession numbers represent sequences generated from this study.

| **Vouchers** | **Geographic details** | **ITS2** | ***trnL-trnF*** | ***trnH-psbA*** | ***rbcL*** | **Determined as** |
| --- | --- | --- | --- | --- | --- | --- |
| BPL2 | Thailand, Loei | **OQ708374** | **OQ834081** | **OQ834052** | **OQ834131** | *Aneura pinguis* |
| BPL6 | Thailand, Loei | **OQ708373** | **OQ834103** | **OQ834078** | **OQ834111** | *Calycularia crispula* |
| Crandall-Stotler 4581 | New Zealand, South Island, Arthur's Pass | DQ98614 | AY50755 | AY50750 | AY50742 | *Lobatiriccardia lobata* |
| JSKB01 | Thailand, Krabi | **-** | **OQ834082** | **OQ834053** | **OQ834114** | *Aneura pinguis* |
| JSPSU01 | Thailand, Songkhla | **OQ708372** | **OQ834083** | **OQ834054** | **OQ834138** | *Aneura pinguis* |
| NA19 | Thailand, Nakhon Nayok | **OQ708371** | **OQ834084** | **OQ834055** | **OQ834128** | *Aneura pinguis* |
| NA20 | Thailand, Nakhon Nayok | **OQ708370** | **OQ834085** | **OQ834056** | **OQ834129** | *Aneura pinguis* |
| NA24 | Thailand, Chiangmai | **OQ708369** | **OQ834086** | **-** | **OQ834140** | *Aneura pinguis* |
| NA25 | Thailand, Chiangmai | **OQ708368** | **-** | **OQ834057** | **OQ834142** | *Aneura pinguis* |
| NA26 | Thailand, Chiangmai | **OQ708367** | **OQ834087** | **OQ834058** | **OQ834123** | *Aneura pinguis* |
| NA27 | Thailand, Chiangmai | **OQ708366** | **-** | **OQ834059** | **OQ834143** | *Aneura pinguis* |
| NA28 | Thailand, Chiangmai | **OQ708365** | **-** | **OQ834060** | **OQ834144** | *Aneura pinguis* |
| NA29 | Thailand, Chiangmai | **OQ708364** | **OQ834088** | **OQ834061** | **OQ834137** | *Aneura pinguis* |
| NA30 | Thailand, Chiangmai | **OQ708363** | **OQ834089** | **OQ834062** | **OQ834135** | *Aneura pinguis* |
| NA31 | Thailand, Chiangmai | **OQ708362** | **-** | **OQ834063** | **OQ834145** | *Aneura pinguis* |
| NA32 | Thailand, Chiangmai | **-** | **OQ834109** | **OQ834080** | **OQ834115** | *Riccardia sp.* |
| NA44 | Thailand, Chiangmai | **OQ708361** | **OQ834104** | **OQ834079** | **OQ834110** | *Calycularia crispula* |
| NA46 | Thailand, Nakhon Nayok | **OQ708360** | **OQ834090** | **OQ834064** | **OQ834125** | *Aneura pinguis* |
| NA47 | Thailand, Nakhon Nayok | **OQ708359** | **OQ834091** | **OQ834065** | **OQ834126** | *Aneura pinguis* |
| NA48 | Thailand, Nakhon Nayok | **OQ708358** | **OQ834092** | **OQ834066** | **OQ834127** | *Aneura pinguis* |
| NA49 | Thailand, Nakhon Nayok | **OQ708357** | **OQ834093** | **OQ834067** | **OQ834124** | *Aneura pinguis* |
| NA57 | Thailand, Chiangmai | **-** | **-** | **OQ834068** | **OQ834121** | *Aneura pinguis* |
| NAPSU01 | Thailand, Songkhla | **-** | **OQ834094** | **OQ834069** | **OQ834139** | *Aneura* sp. |
| NS02 | Thailand, Loei | **OQ708356** | **OQ834095** | **OQ834070** | **OQ834141** | *Aneura maxima* |
| NS011 | Thailand, Loei | **OQ708355** | **OQ834096** | **-** | **OQ834130** | *Aneura pinguis* |
| PN1936 | Thailand, Chiangmai | **-** | **OQ834097** | **-** | **OQ834122** | *Aneura pinguis* |
| PN5206 | Thailand, Chiangmai | **-** | **-** | **OQ834071** | **OQ834112** | *Aneura* sp. |
| PNB10.67 | Indonesia, West Java and Banten | **-** | **OQ834106** | **-** | **OQ834119** | *Aneura maxima* |
| PTEK01 | Thailand, Sakon Nakhon | **-** | **OQ834098** | **OQ834072** | **OQ834113** | *Aneura pinguis* |
| SCAC318 | Thailand, Yala | **-** | **OQ834105** | **-** | **OQ834118** | *Lobatiriccardia coronopus* |
| SCOS412 | Thailand, Phatthalung | **-** | **OQ834107** | **-** | **OQ834120** | Aneuraceae |
| SCOS1077 | Thailand, Phatthalung | **-** | **-** | **OQ834073** | **OQ834116** | Aneuraceae |
| SCOS1091 | Thailand, Phatthalung | **-** | **OQ834108** | **-** | **OQ834117** | *Aneura* sp. |
| SCOS1373 | Thailand, Nakhon Si Thammarat | **-** | **OQ834099** | **OQ834074** | **OQ834133** | *Aneura* sp. |
| SCOS1570 | Thailand, Nakhon Si Thammarat | **-** | **OQ834100** | **OQ834075** | **OQ834136** | *Aneura maxima* |
| SCOS2695 | Thailand, Loei | **OQ708354** | **OQ834101** | **OQ834076** | **OQ834134** | *Aneura maxima* |
| SCOS2701 | Thailand, Loei | **-** | **OQ834102** | **OQ834077** | **OQ834132** | *Aneura* sp. |
| POZW 39872 | E Poland, Białowieza Forest, Zebra Zubra track | KY705516 | KY706039 | KY705726 | KY705826 | *Aneura maxima* |
| POZW 39882 | E Poland, Białowieza Forest, Wysokie Bagno Reserve | KY705517 | KY706040 | KY705727 | KY705827 | *Aneura maxima* |
| POZW 40163 | United Kingdom, Scotland, North Ebudes, coll. D.G. Long | KY705468 | KY705978 | KY705689 | KY705788 | *Aneura pinguis* |
| POZW 40238 | United Kingdom, Ireland, West Galway, coll. D.G. Long | KY705469 | KY705979 | KY705690 | KY705789 | *Aneura pinguis* |
| POZW 40265 | Central Poland, Wielkopolska, valley of Rurzyca river | KY705520 | KY706043 | KY705730 | KY705830 | *Aneura maxima* |
| POZW 40511 | Japan, Mount Lide, N slope | KY705514 | KY706037 | KY705724 | KY705824 | *Aneura pinguis* |
| POZW 40538 | S Poland, Tatry Mts, Wielka Sucha Dolina valley | KY705444 | KY705953 | KY705672 | KY705770 | *Aneura pinguis* |
| POZW 40543 | S Poland, Tatry Mts, Dolina Białki valley | KY705525 | KY706048 | KY705734 | KY705835 | *Aneura maxima* |
| POZW 40544 | Japan, Mount Lide, N slope | KY705513 | KY706036 | KY705723 | KY705823 | *Aneura pinguis* |
| POZW 41409 | NW Poland, Western Pomerania, Słupia river | KY705519 | KY706042 | KY705729 | KY705829 | *Aneura maxima* |
| POZW 42019 | Central Poland, Wielkopolska, SW part of Poznan | KY705438 | KY705945 | KY705667 | KY705764 | *Aneura pinguis* |
| POZW 42133 | S Poland, Pieniny Mts, tributary of Potok Skalskie stream | KY705425 | KY705926 | KY705659 | KY705751 | *Aneura pinguis* |
| POZW 42240 | NW Poland, Western Pomerania, Lake Duze Witno | KY705500 | KY706021 | KY705711 | KY705812 | *Aneura pinguis* |
| POZW 42438 | S Poland, Gorce Mts, Działy Orawskie | KY705528 | KY706051 | KY705736 | KY705838 | *Aneura maxima* |
| POZW 42486 | S Poland, Beskidy Mts, Szczawa stream | KY705413 | KY705912 | KY705649 | KY705740 | *Aneura pinguis* |
| POZW 42747 | Central Poland, Wielkopolska, valley of Rurzyca river | KY705456 | KY705966 | KY705681 | KY705778 | *Aneura pinguis* |
| POZW 42755 | S Poland, Slask, near Katowice | KY705506 | KY706029 | KY705716 | KY705818 | *Aneura pinguis* |
| POZW 42760 | NW Poland, Western Pomerania, Lake Małe Oczko | KY705509 | KY706032 | KY705719 | KY705820 | *Aneura pinguis* |
| POZW 42762 | NW Poland, Western Pomerania, Słupia river | KY705448 | KY705958 | KY705676 | KY705774 | *Aneura pinguis* |
| POZW 42769 | NW Poland, Western Pomerania, Lake Ksiaze | KY705447 | KY705957 | KY705675 | KY705773 | *Aneura pinguis* |
| POZW 42793 | SE Poland, Bieszczady Mts, valley of Beskidnik stream | KY705441 | KY705948 | KY705669 | KY705767 | *Aneura pinguis* |
| POZW 42798 | SE Poland, Bieszczady Mts, valley of Terebowiec stream | KY705411 | KY705910 | KY705647 | KY705738 | *Aneura pinguis* |
| POZW 42820 | S Poland, Beskidy Mts, valley of Kozłecki stream | KY705524 | KY706047 | KY705733 | KY705834 | *Aneura maxima* |
| POZW 42821 | S Poland, Tatry Mts, valley of Biały Potok stream | KY705431 | KY705932 | KY705662 | KY705757 | *Aneura pinguis* |
| POZW 42824 | S Poland, Tatry Mts, Wielka Sucha Dolina valley | KY705476 | KY705986 | KY705696 | KY705792 | *Aneura pinguis* |
| POZW 42861 | S Poland, Pieniny Mts, Limbargowy Potok stream | KY705415 | KY705915 | KY705650 | KY705742 | *Aneura pinguis* |
| POZW 42862 | S Poland, Tatry Mts, NE slope of Skupniów Upłaz Mt. | KY705419 | KY705919 | KY705653 | KY705746 | *Aneura pinguis* |
| POZW 42863 | S Poland, Beskidy Mts, Wygon stream | KY705421 | KY705921 | KY705655 | KY705748 | *Aneura pinguis* |
| POZW 42869 | S Poland, Tatry Mts, Strazyska Valley | KY705435 | KY705936 | KY705664 | KY705761 | *Aneura pinguis* |
| POZW 42879 | SE Poland, Bieszczady Mts, Ustrzyki Górne, small stream | KY705466 | KY705976 | KY705687 | KY705786 | *Aneura pinguis* |
| POZW 42885 | S Poland, Tatry Mts, valley of Biały Potok stream | KY705482 | KY705992 | KY705699 | KY705797 | *Aneura pinguis* |
| POZW 42891 | SE Poland, Bieszczady Mts, tributary of Wołosaty stream | KY705491 | KY706009 | KY705706 | KY705805 | *Aneura pinguis* |
| POZW 42894 | S Poland, Beskidy Mts, valley of Kozłecki stream | KY705496 | KY706014 | KY705709 | KY705810 | *Aneura pinguis* |
| POZW 42896 | SE Poland, Bieszczady Mts, Beskid Pass | KY705503 | KY706024 | KY705713 | KY705815 | *Aneura pinguis* |
| POZW 42898 | Central Poland, Wielkopolska, valley of Rurzyca river | KY705505 | KY706028 | KY705715 | KY705817 | *Aneura pinguis* |
| POZW 42901 | SE Poland, Bieszczady Mts, Old quarry near Brzegi Górne | KY705488 | KY706006 | KY705704 | KY705802 | *Aneura pinguis* |
| POZW 42902 | SE Poland, Bieszczady Mts, valley of Terebowiec stream | KY705522 | KY706045 | KY705732 | KY705832 | *Aneura maxima* |
| POZW 42905 | S Poland, Pieniny Mts, Pieninski Potok stream | KY705422 | KY705923 | KY705656 | KY705749 | *Aneura pinguis* |
| POZW 42908 | S Poland, Tatry Mts, Panszczyca Valley | KY705462 | KY705972 | KY705685 | KY705784 | *Aneura pinguis* |
| POZW 42910 | S Poland, Pieniny Mts, Kotłowy Potok stream | KY705497 | KY706015 | KY705710 | KY705811 | *Aneura pinguis* |
| POZW 42913 | NW Poland, Western Pomerania, Lake Duze Witno | KY705511 | KY706034 | KY705721 | KY705822 | *Aneura pinguis* |
